# PTSD and Complex PTSD: ICD-11 updates on concept and measurement in the UK, USA, Germany and Lithuania

**DOI:** 10.1080/20008198.2017.1418103

**Published:** 2018-01-15

**Authors:** Thanos Karatzias, Marylene Cloitre, Andreas Maercker, Evaldas Kazlauskas, Mark Shevlin, Philip Hyland, Jonathan I. Bisson, Neil P. Roberts, Chris R. Brewin

**Affiliations:** ^a^ School of Health & Social Care, Edinburgh Napier University, Edinburgh, UK; ^b^ NHS Lothian, Rivers Centre for Traumatic Stress, Edinburgh, UK; ^c^ School of Medicine, New York University, USA; ^d^ National Center for PTSD, Veterans Affairs Palo Alto Health Care System, Palo Alto, CA, USA; ^e^ Department of Psychology, Psychopathology and Clinical Interventions, University of Zurich, Zurich, Switzerland; ^f^ Department of Clinical & Organizational Psychology, Vilnius University, Vilnius, Lithuania; ^g^ School of Psychology, Ulster University, Derry, UK; ^h^ School of Business, National College of Ireland, Dublin, Ireland; ^i^ School of Medicine, Cardiff University, Cardiff, UK; ^j^ Psychology and Counselling Directorate, Cardiff and Vale University Health Board, Cardiff, UK; ^k^ Clinical, Education & Health Psychology, University College London, London, UK

**Keywords:** PTSD, CPTSD, ITQ, prevalence, ICD-11, TEPT, TEPT-C, ITQ, prevalencia, CIE-11, PTSD, CPTSD, ITQ, 流行, ICD－11, • Preliminary findings suggest that the ITQ is a reliable and valid instrument for the assessment of PTSD and CPTSD as per ICD-11 proposals.• CPTSD is common in clinical samples and populations samples across four countries.• Further cross-cultural work on CPTSD is proposed.

## Abstract

The 11th revision to the World Health Organization’s International Classification of Diseases (ICD-11) proposes two distinct sibling conditions: Posttraumatic Stress Disorder (PTSD) and Complex PTSD (CPTSD). In this paper, we aim to provide an update on the latest research regarding the conceptual structure and measurement of PTSD and CPTSD using the International Trauma Questionnaire (ITQ) as per ICD-11 proposals in the USA, UK, Germany and Lithuania. Preliminary findings suggest that CPTSD is common in clinical and population samples, although there may be variations across countries in prevalence rates. In clinical samples, preliminary evidence suggests that CPTSD is a more commonly observed condition than PTSD. Preliminary evidence also suggests that the ITQ scores are reliable and valid and can adequately distinguish between PTSD and CPTSD. Further cross-cultural work is proposed to explore differences in PTSD and CPTSD across different countries with regard to prevalence, incidence, and predictors of PTSD and CPTSD.

## Abstract (es)

La 11ª revisión de la Clasificación Internacional de Enfermedades (CIE-11) de la Organización Mundial de la Salud propone dos afecciones hermanas distintas, el trastorno por estrés postraumático (TEPT) y el TEPT Complejo (TEPT-C). En este artículo, nuestro objetivo es proporcionar una actualización sobre las últimas investigaciones relacionadas con la estructura conceptual y la medición de TEPT y TEPT-C utilizando el Cuestionario Internacional de Trauma (ITQ, siglas en inglés de International Trauma Questionnaire) según las propuestas de la CIE-11 en los Estados Unidos, el Reino Unido, Alemania y Lituania. Los hallazgos preliminares sugieren que el TEPT-C es frecuente en muestras clínicas y de población, aunque puede haber variaciones entre países en las tasas de prevalencia. En muestras clínicas, la evidencia preliminar sugiere que el TEPT-C es una condición más comúnmente observada que el TEPT. La evidencia preliminar también sugiere que las puntuaciones del ITQ son fiables y válidas y pueden distinguir adecuadamente entre TEPT y TEPT-C Se propone un trabajo intercultural adicional para explorar las diferencias en el TEPT y el TEPT-C en los diferentes países con respecto a la prevalencia, la incidencia y los predictores del TEPT y el TEPT-C

## Abstract (zh)

世界卫生组织发布的第11版国际疾病分类（ICD－11）提出了两种不同但相仿的疾病：创伤后应激障碍（PTSD）和复杂PTSD（CPTSD）。在本文中，我们目标是更新关于 PTSD和 CPTSD 在概念结构和测量上的研究进展。我们在英国、美国、德国和立陶宛根据 ICD-11使用国际创伤问卷（ITQ）。初步研究发现显示 CPTSD 在临床和普通人群中普遍存在，尽管在不同国家之间的流行率可能有所不同。在临床样本中，初步证据显示 CPTSD 比 PTSD 更加常见，同时也支持ITQ 分数可以有效可靠地区分 PTSD 和 CPTSD。未来还需要横向研究探索 PTSD 和 CPTSD 在不同国家之间的流行率、发生率和预测指标的区别。

## 1. Introduction

The upcoming 11th revision to the World Health Organization’s International Classification of Diseases (ICD-11), to be published in 2018, proposes two distinct sibling conditions, Posttraumatic Stress Disorder (PTSD) and Complex PTSD (CPTSD), under a general parent category of ‘Disorders specifically associated with stress’. PTSD is comprised of three symptom clusters including: (1) re-experiencing of the trauma in the here and now (Re), (2) avoidance of traumatic reminders (Av), and (3) a persistent sense of current threat that is manifested by exaggerated startle and hypervigilance (Th). ICD-11 CPTSD includes the three PTSD clusters and three additional clusters that reflect ‘disturbances in self-organization’ (DSO): (1) affective dysregulation (AD), (2) negative self-concept (NSC), and (3) disturbances in relationships (DR) (Maercker et al., 2013). These disturbances are proposed to be typically associated with sustained, repeated, or multiple forms of traumatic exposure (e.g. genocide campaigns, childhood sexual abuse, child soldiering, severe domestic violence, torture, or slavery), reflecting loss of emotional, psychological, and social resources under conditions of prolonged adversity (Cloitre, Garvert, Brewin, Bryant & Maercker, 2013).

The qualitative distinction between PTSD and CPTSD symptomatology has been supported in different trauma samples including those experiencing interpersonal violence (Cloitre et al., 2013), rape, domestic violence, traumatic bereavement (Elklit, Hyland, & Shevlin, 2014), and victims of institutional abuse such as that occurring within foster care and religious organizations (Knefel, Garvert, Cloitre, & Lueger-Schuster, 2015). Samples have also included young adults (Perkonigg, Hoffler, Wittchen, Trautmann, & Maercker, 2014) and children (Sachser, Keller, & Goldbeck, 2016). The proposed three-factor structure of ICD-11 PTSD (Re, Av, Th) has been supported in a number of studies (e.g. Gluck, Knefel, Tran, & Lueger-Schuster, 2016; Hansen, Hyland, Armour, Shevlin, & Elklit, 2015; Tay et al., 2016). In addition, the second-order factorial structure of CPTSD in which the disorder is comprised of both PTSD and DSO has also been supported (e.g. Hyland et al., 2017b, 2017c; Shevlin et al., 2017).

In this paper, we aim to provide an update on the latest research regarding the conceptual structure and measurement of PTSD and CPTSD using the International Trauma Questionnaire (ITQ) as per ICD-11 proposals in the USA, UK, Germany, and Lithuania.

### 1.1. The International Trauma Questionnaire (ITQ)

The ITQ (Cloitre, Roberts, Bisson, & Brewin, 2017) is still under development and is a self-report measure that was developed for the assessment of ICD-11 PTSD and CPTSD diagnoses. In its current form, the ITQ is a 23-item self-report measure with seven PTSD and 16 DSO items. It was previously called the ICD-11 Trauma Questionnaire (ICD-TQ). Three items are used to measure Re (items P1–P3), two items to measure Av (items P4–P5), and two items to measure Th (items P6–P7). Sixteen items represent the three clusters of AD (items C1–C9), NSC (items C10–C13), and DR (items C14–C16). Symptom endorsement for all items is scored on a Likert scale ranging from 0 (‘not at all’) to 4 (‘extremely’). The PTSD items are answered in response to the question ‘how much have you been bothered by that problem for the past month?’ and the DSO items are answered in terms of how one ‘typically feels, thinks about themselves, or relates to others’. A diagnosis of PTSD requires that: (i) an individual has experienced a traumatic event, (ii) indicates the presence of at least one symptom in each of its three clusters (as indicated by a score of ≥ 2 on the Likert scale – ‘Moderately’), and (iii) indicates functional impairment associated with these symptoms. A probable diagnosis of CPTSD requires that the PTSD criteria are met and the following scores for each of the three DSO clusters: AD scores ≥ 10 on items C1–C5 (Affective Dysregulation-hyperactivation) or a score of ≥ 8 on items C6–C9 (Affective Dysregulation-hypoactivation); NSC requires a score ≥ 8 on items C10–C13, and DR requires a score ≥ 6 on items C14–C16.

All studies to date have used at least two Re-experiencing items (Re1: Upsetting dreams, and Re2: Reliving the event in the here and now). A third Re item (Re3: Feeling very upset when something reminded you of the experience) was also included in some studies. This is currently under consideration for use with traumatized individuals who possess no clear memory of their index trauma (e.g. possibly due to childhood traumatization or traumatic brain injuries). The addition of Re3 tends to increase the percentage of participants who meet the diagnostic criteria for this cluster but does not dramatically change overall PTSD rates. The process of reducing the DSO indicators to two per cluster is currently underway.

### 1.2. Initial standardization of the ITQ in UK and Lithuania

**Figure 1.**
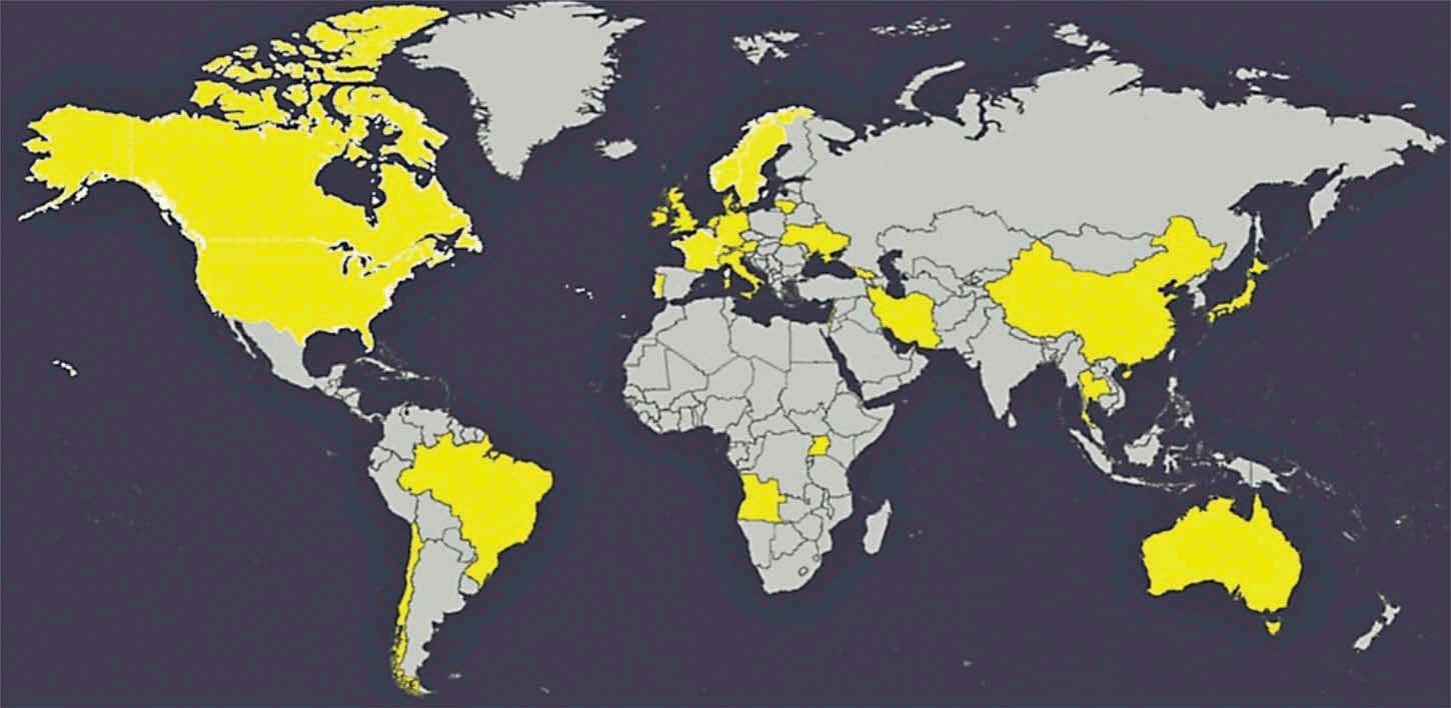
Countries in which the ITQ has been used: Angola, Australia, Austria, Brazil, Canada, Chile, China, Denmark, France, Georgia, Germany, Hong Kong, Iran, Ireland, Israel, Italy, Japan, Lebanon, Lithuania, Netherlands, Norway, Portugal, Sweden, Switzerland, Thailand, Uganda, UK, Ukraine, USA.

Numerous projects are currently taking place worldwide for the standardization of the ITQ. The ITQ has been used, or is currently in use, in 29 countries across six continents (see Figure 1).

Results from relevant projects in the UK and Lithuania are presented as follows. The ITQ has been initially validated in a sample of individuals who were referred for psychological therapy to a National Health Service (NHS) trauma centre in Scotland (*N* = 193) (Karatzias et al., 2016, 2017). In this project, participants completed the ITQ and measures of traumatic life events, DSM-5 PTSD, emotion dysregulation, self–esteem, and interpersonal difficulties. Using the ITQ, two subgroups of treatment-seeking individuals could be empirically distinguished based on different patterns of symptom endorsement: a small group high in PTSD symptoms only (24%) and a larger group high in CPTSD symptoms (76%) (Karatzias et al., 2017). Confirmatory factor analysis (CFA) results supported the factorial validity of the ITQ with results in line with ICD-11 proposals. The ITQ demonstrated satisfactory internal reliability, and correlation results indicated that the scale exhibited convergent and discriminant validity. CPTSD was more strongly associated than PTSD with more frequent trauma, a greater accumulation of different types of childhood traumatic experiences, and higher levels of functional impairment (Karatzias et al., 2016).

The ITQ has been further validated using a second British clinical sample from Wales (*N* = 171) (Hyland et al., 2017b). This study evaluated the ITQ in comparison to DSM-5 PTSD, generalized anxiety disorder, depression, negative self-beliefs, negative beliefs about the world, and distress tolerance. Findings indicated a lower probable prevalence rate for ICD11 PTSD/CPTSD than for DSM-5 PTSD. CFA results replicated the findings of Karatzias et al. (2016), and the ICD-11 PTSD and DSO factors differentially predicted multiple psychological variables.

Validation of ICD-11 proposals for PTSD and CPTSD, as well as of the ITQ, is particularly relevant in Lithuania as findings regarding PTSD prevalence in that country are ambiguous. General population studies reveal high levels of trauma exposure (Kazlauskas & Zelviene, 2016), although recent analyses of health care data showed that only about 1% of probable PTSD cases are diagnosed (Kazlauskas, Zelviene, & Eimontas, 2017). Problems in diagnosing stress-related disorders could be attributed to lack of health care resources (Kazlauskas & Zelviene, 2016), and to limited knowledge about PTSD among health professionals. However, underdiagnosing of PTSD in Lithuania could also indicate a different symptom profile in the Lithuanian population exemplifying the need for cross-cultural validation of ICD-11 proposals.

The ICD-11 PTSD and CPTSD structures were validated recently in a clinical treatment-seeking sample (*n* = 280) using a Lithuanian version of the ITQ (Kazlauskas, Gegieckaite, Hyland, Zelviene, & Cloitre, in press). CFA supported the factor structure of ICD-11 PTSD and CPTSD. The best fitting factor analytic model was a two-factor (PTSD and DSO) second-order model consistent with the findings of Karatzias et al. (2016) and Hyland et al. (2017b) in their studies with two UK clinical samples. Kazlauskas et al. also conducted an LCA and identified three unique classes of trauma survivors whose patterns of symptom endorsements reflected a PTSD class, a CPTSD class, and a low symptom class. Further epidemiological studies of ICD-11 PTSD and CPTSD in the general population of Lithuania, as well as studies of predictors of PTSD and CPTSD, are required.

### 1.3. PTSD and CPTSD in population samples in Germany

An ongoing project in Germany surveyed 2524 individuals from the general population. A main feature of this study was the assessment of one-month prevalence in contrast to lifetime prevalence rates. This was in accordance with a previous study in the same setting investigating PTSD incidence according to DSM-IV. In this previous study, an overall one-month prevalence rate of 2.3% was found (with similar rates for women and men: 2.5% and 2.1%, respectively) and with an age-related increase up to 3.4% for the group between 60 to 95 years of age (Maercker, Forstmeier, Wagner, Glaesmer, & Brähler, 2008). This rise can most likely be explained by experiences related to World War II (cf. Burri & Maercker, 2014)

For the current report, only methodological aspects can be discussed since results have been submitted for publication elsewhere (Maercker, Hecker, Augsburger & Kliem, in press). Households in Germany (*n* = 3416) were sampled according to representative country regions, and members of those households were randomly selected applying the Kish selection grid method. The ITQ version 1.4 was used. The ITQ presented with good psychometric properties with reliability coefficients ranging between α = .63 (avoidance subscale) to α = .91 (total scale) for the PTSD clusters and between α = .73 (Affective Dysregulation-hyperactivation subscale) to α = .91 (total scale) for the CPTSD clusters. Furthermore, lifetime experience of eight different potentially traumatic events were assessed, and participants were asked to indicate their worst subjective event. The data generated one-month prevalence rates for PTSD, CPTSD, and a clinical variant of CPTSD according to ICD-11 criteria. Moreover, conditional prevalence and differential predictors of PTSD and CPTSD were calculated. Taken together, combined prevalence rates for the sibling diagnoses of PTSD and CPTSD were in the range of previously reported results. However, rates were lower than in similar studies from the US. This finding might be explained because exposure to potentially traumatic events is less likely in European countries compared to the US (see Burri & Maercker, 2014). In accordance with the preceding study (Maercker et al., 2008), no gender differences were observed although the effect of an age-related increase could not be replicated in the current study. It might well be the case that individuals with World War II related experiences might have passed away during the interval of the two studies. Finally, results point to the importance of sexual violence experiences in differentiating between PTSD and CPTSD, with sexual violence more likely associated with CPTSD (Hyland et al., 2017a).

## 2. ICD-11 PTSD and CPTSD prevalence rates and correlates in a USA sample

The ITQ was assessed in a nationally representative household sample of adults (*n* = 1893) in the USA. Data were collected using an existing online research panel that was randomly recruited through probability based sampling. Inclusion criteria were that the respondents be aged 18–70 and have experienced at least one traumatic event in their lifetime. Latent class analyses (LCA) identified distinct PTSD and CPTSD groups within the population supporting the construct validity of CPTSD and CFA results supported the factorial validity of the ITQ with results in line with ICD-11 proposals. These results will be submitted for publication elsewhere.

In this report, we present preliminary analyses on the lifetime (not current) prevalence rates and correlates of PTSD and CPTSD as determined by the ITQ. The combined lifetime prevalence of PTSD and CPTSD was 7.3%. The lifetime prevalence rate of PTSD was 4.0% and that of CPTSD was 3.3%. These lifetime rates are quite similar to those found for PTSD in other nationally representative US prevalence studies: the National Comorbidity Study (NCS) reported a lifetime PTSD estimate of 7.8% (Kessler, Sonnega, Bromet, Hughes, & Nelson, 1995) and the National Comorbidity Study replication (NCS-R) reported a lifetime estimate of 6.8% (Kessler, Chiu, Demler, & Walters, 2005).

Women compared to men were more than twice as likely to meet criteria for PTSD and for CPTSD. Cumulative childhood interpersonal violence was as a stronger predictor of CPTSD than of PTSD. CPTSD was associated with greater comorbid symptom burden and substantially lower psychological well-being, suggesting the greater severity of the disorder. Patterns of symptom endorsement across the six symptom clusters comprising CPTSD revealed that symptom endorsement rates for PTSD and DSO were equally high suggesting that the two components of the disorder are equally salient. Among the DSO symptom clusters, the most frequently endorsed cluster was negative self-concept, suggesting the critical role problems in self-concept may play in CPTSD.

## 3. Conclusions

Preliminary findings suggest that CPTSD is common in clinical and general population samples although there may be variations across countries in prevalence rates. In clinical samples of trauma victims, preliminary evidence suggests that CPTSD is a more common condition than PTSD. Preliminary findings also suggest that CPTSD is a more debilitating condition compared to PTSD with regard to survivors’ functioning. Childhood, multiple, and interpersonal trauma are all most likely associated with CPTSD as opposed to PTSD in both clinical and population samples.

Furthermore, preliminary evidence suggests that the ITQ is an instrument that produces reliable and valid scores and can adequately distinguish between PTSD and CPTSD. Current results provide initial support for the psychometric properties of the 23-item initial version of the ITQ. Future theoretical and empirical work will be required to generate a final version of the ITQ that will match the diagnostic structure of PTSD and CPTSD and be standardized in several cultures and languages. In particular, we aim to further improve clinical utility by reducing the current list of symptoms in the DSO cluster, especially so for AD. The reduction in the number of DSO items is to align with the overall goals of the ICD-11 that diagnoses maximize clinical utility and are described using as few a number of symptoms as is possible. There is also further work to be done on test-rest reliability as well as the discriminant validity of the ITQ, and exploring further the relationship between PTSD and CPTSD and depression, anxiety, and substance misuse disorders. This work must be replicated in various countries and cultures as there have been concerns about the validity of traumatic stress disorders as culturally bound conditions (Hinton & Lewis-Fernández, 2011). Cross-cultural work is required to explore differences in PTSD and CPTSD across different countries with regard to prevalence, incidence, and predictors of PTSD and CPTSD as per ICD-11 proposals. This work is essential to enhance the cross-cultural applicability of the new condition of CPTSD. Finally, in addition to the development and validation of the ITQ, work is also underway on the development a clinician-administered diagnostic interview for PTSD and CPTSD. This measure is called the International Trauma Interview (ITI; Roberts, Cloitre, Bisson, & Brewin, 2017). The development of a diagnostic interview is a critical element of ongoing efforts to assess the validity of the ICD-11 proposals, as well as to determine the effectiveness of the ITQ in identifying clinical cases.
